# Evolution of Anti-B Cell Therapeutics in Autoimmune Neurological Diseases

**DOI:** 10.1007/s13311-022-01196-w

**Published:** 2022-02-18

**Authors:** Panos Stathopoulos, Marinos C. Dalakas

**Affiliations:** 1 1st Department of Neurology, National and Kapodistrian University of Athens, Athens, Greece; 2 Thomas Jefferson University, Philadelphia, PA USA; 3 Neuroimmunology Unit, National and Kapodistrian University of Athens, Athens, Greece

**Keywords:** B cells, Autoimmunity, Neurological disorders, Autoantibodies, Rituximab, Monoclonal antibodies

## Abstract

B cells have an ever-increasing role in the etiopathology of a number of autoimmune neurological disorders, acting as antigen-presenting cells facilitating antibody production but also as sensors, coordinators, and regulators of the immune response. In particular, B cells can regulate the T cell activation process through their participation in antigen presentation, production of proinflammatory cytokines (bystander activation or suppression), and contribution to ectopic lymphoid aggregates. Such an important interplay between B and T cells makes therapeutic depletion of B cells an attractive treatment strategy. The last decade, anti-B cell therapies using monoclonal antibodies against B cell surface molecules have evolved into a rational approach for successfully treating autoimmune neurological disorders, even when T cells seem to be the main effector cells. The paper summarizes basic aspects of B cell biology, discusses the roles of B cells in neurological autoimmunities, and highlights how the currently available or under development anti-B cell therapeutics exert their action in the wide spectrum and immunologically diverse neurological disorders. The efficacy of the various anti-B cell therapies and practical issues on induction and maintenance therapy is specifically detailed for the treatment of patients with multiple sclerosis, neuromyelitis-spectrum disorders, autoimmune encephalitis and hyperexcitability CNS disorders, autoimmune neuropathies, myasthenia gravis, and inflammatory myopathies. The success of anti-B cell therapies in inducing long-term remission in IgG4 neuroautoimmunities is also highlighted pointing out potential biomarkers for follow-up infusions.

**Supplementary Information:**

The online version contains supplementary material available at 10.1007/s13311-022-01196-w.

## Introduction

Autoimmunity is the non-physiological state where immune components exert their actions against self. Similar to systemic autoimmune diseases, autoimmune neurological diseases may be mediated by all elements of the immune system including B cells. Traditionally, most of the work on autoimmune neurological disorders has been centered on the role of T cell subtypes because investigators had focused on multiple sclerosis, the commonest neuroimmunological disorder, and its experimental model experimental allergic encephalomyelitis (EAE), which is predominately mediated by effector T cells; this is also the case for its peripheral counterpart experimental allergic neuritis. In the past few years, however, these views have changed and the role of B cells, not only as antibody-producing cells but also as sensors, coordinators, and regulators of the immune response, has strongly emerged generating significant clinical and research interest. It has now become evident that B cells play a fundamental role in the pathogenesis not only of demyelinating diseases but also in other autoimmune CNS and PNS diseases like encephalopathies, peripheral neuropathies, neuromuscular junction disorders, and muscle diseases. A major relevant development in the field has been the availability of new biological agents targeting B cells or B cell pathways, highlighting the role of B cell autoimmunity in the pathophysiology of neurological disorders and offering exciting new therapeutic interventions.

This paper provides a brief overview of B cell biology, addresses the role of B cells in autoimmune neurological disorders, and discusses the anti-B cell agents, either currently on the market and most of them approved for the treatment of autoimmune neurological disorders, or in ongoing trials. The uniqueness of IgG4-related neuro-autoimmunities and the evolving concept that anti-B cell agents are the most rewarding therapies in providing long-term remissions are specifically highlighted.

## A Personal Historical Perspective on Anti-B Cell Therapy in Neurology

Witnessing the evolution of B cell therapy in neurology has been an impressive success story thanks to the contributions of many esteemed colleagues and friends. When we first reviewed the topic in 2006 for the parent journal of Neurotherapeutics, there was no anti-B cell therapy approved or any controlled study published in any neurological disease; the field was however viewed as highly promising for the future of autoimmune neurology based on small uncontrolled series [1]. It was just 2 years later that ongoing controlled studies with rituximab were discussed [2, 3] with the very first, pioneering controlled study in multiple sclerosis (MS) published the same year by Houser et al. [4]. Since then, the field has progressed with an impressively galloping pace. Even in the previous review on B cell therapies for this journal 5 years ago [5], although several controlled studies had been conducted, there was still no approved anti-B cell agent for neurology. In just 6 years since then, we have now 5 drugs (6 with the anti-complement agent eculizumab) approved for various neurological autoimmune diseases while rituximab, the original anti-CD20 agent and its generic FDA-approved biosimilar (Truxima), dominate the field as the main off-label anti-B cell drugs worldwide. In addition, B cell neuroimmunobiology has dramatically advanced. The paper is not only highlighting this progress but also provides an updated view of the present and the immediately upcoming future.

## Principles of B Cell Development and Maturation

### Early Antigen-Independent B Cell Development

**Fig. 1:**
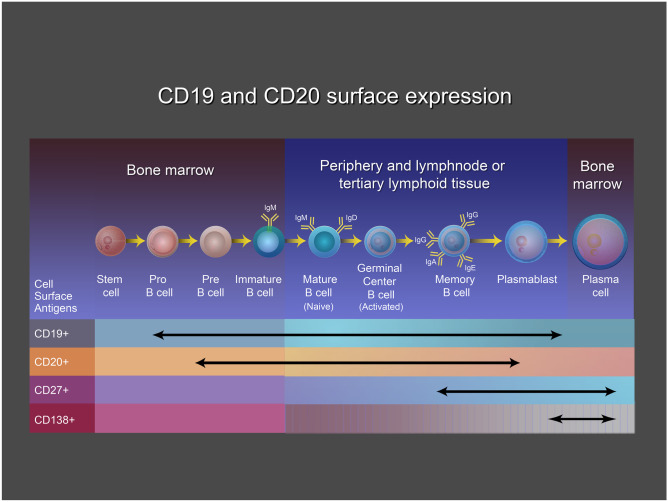
B cell CD markers. The B cell maturation process involves two phases of differentiation—an antigen-independent process in the bone marrow, where V(D)J recombination takes place, and an antigen-dependent process that occurs in secondary and tertiary lymphoid tissue. Specific CD (cluster of differentiation) markers such as CD19, CD20, CD27, and CD138 are helpful for distinguishing different maturation phases, including pro- and pre-B cells, immature and mature naïve B cells, memory B cells, plasmablasts and plasma cells [From Dalakas MC (1)]

In humans, following birth, the majority of B lymphocytes develop from hematopoietic stem cells in the bone marrow. There, during the first stages of B cell development, they obtain their antibody specificity by sequential rearrangement of the immunoglobulin (Ig) heavy (H-chain)- and light-chain (L-chain) V(D)J genes in an antigen-independent manner (Fig. 1) [6, 7]. Once V(D)J recombination is complete and functional IgM molecules are expressed οn the immature B cell surface, these cells exit the bone marrow, enter the peripheral B cell compartment, and migrate to the lymphoid organs where they finalize their development by differentiating from immature B cells to mature naïve follicular or marginal-zone B cells [8]. In parallel, two tolerance checkpoints remove autoreactive B cells and prevent them from entering the peripheral naïve B cell pool; the first is a central tolerance checkpoint that removes the early immature autoreactive B cells in the bone marrow, and the second is a peripheral tolerance checkpoint that removes the new emigrant autoreactive B cells in the periphery [9].

### Mature B Cell Development—Follicular B cells

Mature naïve follicular cells (also called re-circulating B cells) express both IgM and IgD isotypes and have the ability to re-circulate through the follicles of secondary lymphoid organs in search of their cognate antigen. Once they encounter it, the antigen is endocytosed and processed into linear peptides before being displayed on the cell surface by MHC-II molecules [10].

**Fig. 2:**
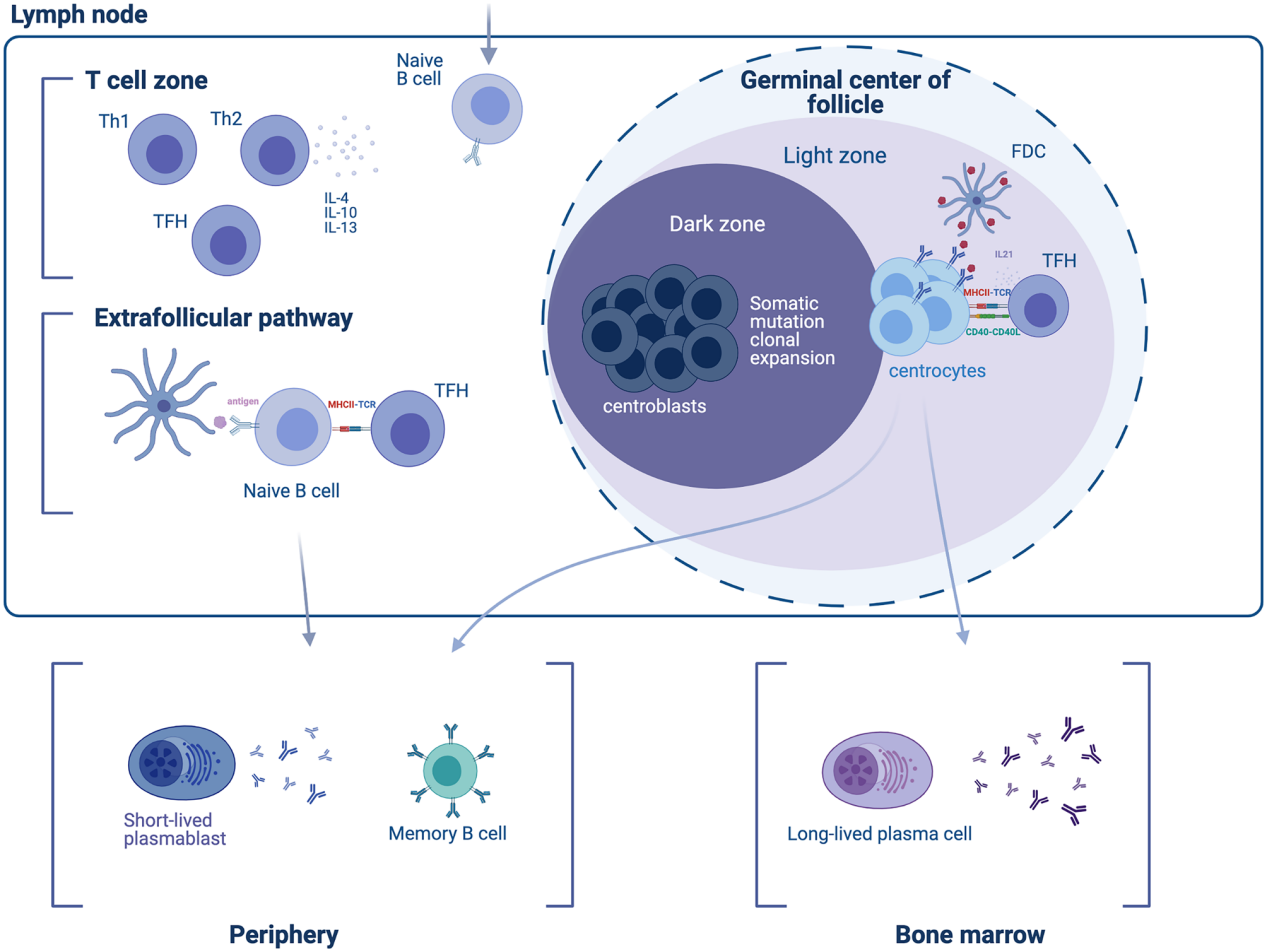
Differentiation of B cells in response to a T cell–dependent antigen. Responding naïve B cells enter the T cell zone of the lymph node (upper left), where their differentiation is facilitated by cytokines and the encounter of the antigen on follicular dendritic cells (FDCs). The antigen activates the B cell receptor, is taken up, and presented to Tfh cells through the B cell MHC-II. This initial, extrafollicular pathway gives rise to short-lived plasmablasts that enter the periphery, and germinal center (GC)–independent memory B cells. In a second phase, activated B cells enter the GC dark zone, where they somatically mutate and clonally expand (therefore termed centroblasts). B cell cycle between the dark and the light zones (where they are termed centrocytes). The dynamic cycle of the GC allows centrocytes that entered the light zone to be chosen based on the affinity of their BCRs to the antigen. Low-affinity B cells that are not presenting antigen on their BCRs will eventually become apoptotic and die. B cells that do present the antigen receive help from Tfh through CD40L and IL21 survival signals. The end-products of the GC reaction are memory B cells, and long-lived plasma cells. GC memory B cells will enter the periphery and re-enter the GC upon BCR stimulation. LLPCs exit the GC and find a survival niche, typically the bone marrow (Figure created with BioRender.com)

At this stage, B cells migrate to extrafollicular spaces and interact with helper T cells (Th) and antigen-presenting dendritic cells (Fig. 2). There, Th cells further activate B cells with high antigen affinity, via CD40L, to differentiate into plasmablasts (short-lived plasma cells) [11, 12]. Plasmablasts are of practical significance in neurologic therapeutics because (a) they produce antibodies that form immune complexes, which are taken up by follicular dendritic cells resulting in the production of chemokines and the attraction of activated B cells back to the follicular space to initiate germinal center formation and (b) they are targeted, either directly or indirectly, by the available anti-B cell monoclonal antibodies (mAbs) suppressing disease activity as discussed later. Previously activated Th cells differentiate further under B cell influence into T follicular helper cells (Tfh) and also migrate towards germinal centers. At this time point, the duration of interaction between Tfh cells and B cells at the B cell/T cell zone border determines the fate of the activated B cells; if it is long enough, they differentiate into germinal center B cells; otherwise, they enter into the germinal center–independent memory B cell pool [13].

### Memory B Cells: Long-Lived Plasma Cells and Plasmablasts

A number of events, highly relevant to neuro-autoimmunity, including affinity maturation, isotype switching, generation of memory B cells, and long-lived plasma cells, take place after a vigorous clonal expansion of activated B cells and germinal center creation at lymphoid follicles. In proliferating germinal centers, B cell affinity maturation through somatic hypermutation of the IgV genes results in the development of B cells with increased antigen affinity. Affinity-matured germinal center B cells terminally differentiate into long-lived, quiescent plasma cells that migrate back to the bone marrow and maintain long-term antibody production [14]. Another fate for affinity-matured germinal center B cells is to exit germinal centers as memory B cells. These antigen-experienced B cells express high-affinity surface antibodies (B cell receptors (BCRs)) and have the ability to quickly differentiate into efficient plasma cells upon future cognate antigen encounters [13]. The involvement of memory B cells in a number of autoimmune disorders has been highlighted by the fact that repopulation of the B cell compartment after B cell depletion with memory B cells (as opposed with transitional, naive B cells) correlates with breakthrough disease activity or reemergence of antibodies, as shown in Myasthenia Gravis [15], anti-MAG neuropathy [16], rheumatoid arthritis (RA), and pemphigus [17–21].

### CD Markers of B Cells at Various Stages of Maturation Relevant to Anti-B Cell Therapies

Specific markers identify the phases of B cell differentiation from stem cells to plasma cells. Typically, CD19 is expressed from the pro-B cell stage till the late plasmablast stage, while CD20 is expressed after the pro-B cell stage. Other important markers are CD27, which is present in most memory B cells (with the exception of double negative CD27-IgD-B cells), late plasmablast and plasma cell stages, and CD138, expressed only on plasma cells (Fig. 1). The presence of these markers is instrumental in understanding the development of B cell–specific and stage-specific therapies, appreciating the duration of a beneficial effect, or serving as potential biomarkers denoting the need for repeat therapy. Memory B cells, plasmablasts, and long-lived plasma cells may also migrate to the brain prompted by specific chemokines, such as CXCL10, CXCL12, and CXCL13, secreted from the endothelial cell wall. Of note, CXCR5 on B cells typically binds CXCL13 expressed in B cell follicles [22].

### B Cell Trophic Factors: BAFF, APRIL, and Their Receptors

BAFF (B cell activating factor of the tumor necrosis factor (TNF) family), also known as BLyS, TALL1, TNFSF13B, has been identified as a factor essential for B cell survival and maturation along with the BCR [23, 24]. APRIL (A ProlifeRation Inducing Ligand), also known as TALL2 and TNFSF13A, is also a member of the TNF family that shares 30% homology with BAFF and has similar functions [25]. BAFF and APRIL are both type II transmembrane proteins cleaved by a furin protease to produce their soluble forms [26, 27]. Major sources of BAFF and APRIL in humans are neutrophils and monocytes (macrophages and dendritic cells) and activated T cells [28–30]. BAFF binds strongly to BAFF-R (BAFF-receptor, also known as TNFRSF13C), to TACI (transmembrane activator and cyclophilin ligand interactor, also known as TNFRSF13B), NGR (Nogo-66 receptor, also known as RTN4R), and weakly to BCMA (B cell maturation antigen, also known as TNFRSF17) [31–33]. In contrast, APRIL binds strongly to BCMA and moderately to TACI [32, 34]. BAFF-R expression is absent during the very early stages of the B cell lineage; its expression coincides with functional BCR expression by immature B cells in the bone marrow and is essential for B cell survival and maturation [23, 24, 35, 36]. TACI is mainly expressed on memory B cells and plasma cells as well as some CD27 cells and BCMA on some activated B cells, plasmablasts, and plasma cells [37–39]. Both TACI and BCMA also exist in soluble forms [40, 41]. Although a number of these molecules have been the target of anti-B cell therapies as discussed later, the results are overall unimpressive or disappointing not only in autoimmune neurological diseases like MS and myasthenia gravis (MG), but also in rheumatologic autoimmunities.

### Regulatory B Cells and B Cell–Related Cytokines

In the last decade, an additional role of B cells as a negative regulator of autoimmune responses has emerged from the study of murine models, and is currently being explored in humans [42, 43]. Unlike regulatory T cells (Tregs), regulatory B cells (Bregs) are not, at least yet, characterized by the expression of a lineage-specific transcription factor (like FOXP3 in Tregs) but rather by their ability to produce a variety of anti-inflammatory signals within an inflammatory environment [44], mainly IL-10 [45]. Interestingly, IL-10 also plays a central role in the IgG4 subclass-switch [46]. In humans, the B cells responsible for anti-inflammatory cytokine production (IL-10) have been found to be mostly naïve B cells [47] expressing CD19, 20, 21, 22, 24, 27, 38, and 40 [48]. In mice, anti-inflammatory IL-10 and IL-35 can be produced by IgM + plasma cells [49] and gut-derived IgA plasma cells that migrate to the brain during EAE ameliorating its severity [50]. Of relevance, stool IgA-bound bacteria are decreased in MS patients during relapse [50]. Notably, memory B cells from MS patients often produce pro-inflammatory cytokines such as lymphotoxin, TNF-α, and GM-CSF [47, 49, 51].

## B Cell Involvement in Neurological Autoimmune Disorders and B Cell–Targeted Therapies

**Fig. 3:**
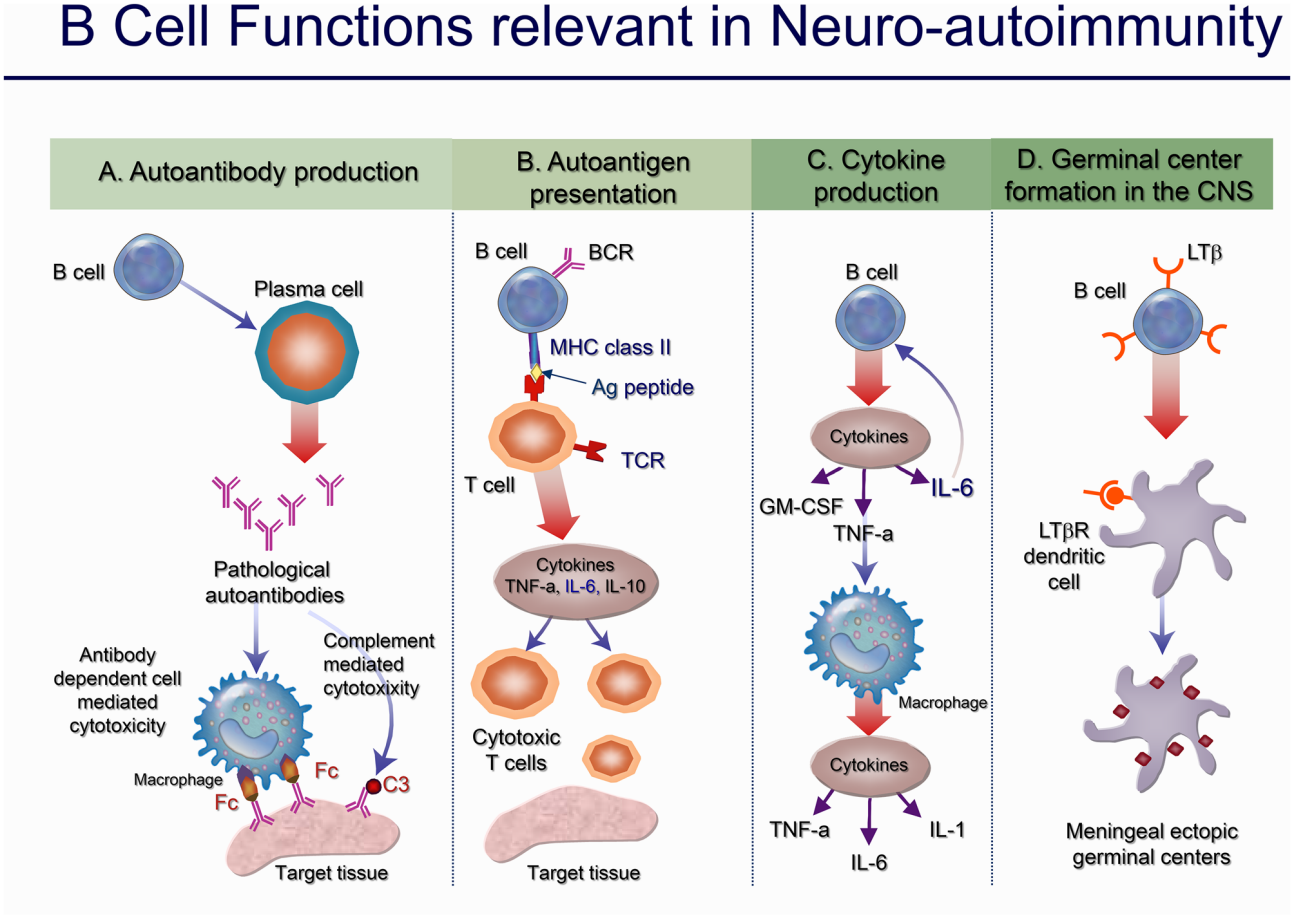
B cell functions in autoimmune neurological disorders. (A) Production of antibodies that cause tissue damage either via complement activation or antibody-dependent cell-mediated cytotoxicity. (B) Antigen presentation, which can result in clonal expansion of cytotoxic T cells and cytokine production. (C) Production of proinflammatory cytokines, such as IL-6, TNF, and GM-CSF, which can activate macrophages and enhance tissue damage. (D) De novo formation and maintenance of ectopic germinal centers in the intermeningeal spaces (neolymphogenesis). Abbreviations: Fc, constant fragment; BCR, B cell receptor; TCR, T cell receptor; MHC, major histocompatibility complex; GM-CSF, granulocyte macrophage-colony-stimulating factor; IL, interleukin; LTβR, lymphotoxin-β receptor; LTβ, lymphotoxin-β; TNF, tumor necrosis factor [from Dalakas MC (1-3]

As mentioned earlier, the main role of B cells in the immune response implicates them in complement activation, antigen presentation, antibody secretion, and cytokine production (Fig. 3) [52]. In autoimmunity, an important effector action of B cells is the production of autoantibodies by plasma cells that have evaded the self-tolerance checkpoints [53]. Although in some neurological diseases autoantibodies against surface antigens are directly pathogenic (e.g. the acetylcholine receptor (AChR) in MG, some synaptic antigens like the N-methyl-d-aspartate receptor (NMDAR) in NMDAR-encephalitis, aquaporin-4 (AQP4) in neuromyelitis optica (NMO)), in others (like those seen in paraneoplastic neuropathies, multifocal motor neuropathy (MMN), or Stiff-Person Syndrome (SPS)), autoantibodies directed against intracellular antigens do not exert a direct pathogenic effect and may only be disease markers. In these cases, either the pathogenic antibody has not yet been identified or it is the antibody-independent functions of B cells, including antigen presentation, co-stimulation, cytokine production, and coordination of T cell functions, that can implicate B cells in the pathogenesis [3]. Accordingly, several agents targeting B cells have been successfully applied in neurological disorders as summarized in Table 1.

**Table 1:** Anti-B cell therapeutics in autoimmune neurological diseases

Agent	Target	Clinical efficacy
		(text in brackets denotes supporting evidence)
Rituximab	CD20	MS (phase II), NMOSD (large series), MOGAD (case series), AE (large series in NMDAR AE)
		MG (large series), Ab-mediated CIDP (series), DM (phase II), PM (phase II),
		(SPS and MAG neuropathy: controlled studies did not reach significance but clinical data support efficacy in 40% of patients)
Ocrelizumab	CD20	MS (FDA-approved for MS)
Ofatumumab	CD20	MS (FDA-approved for MS)
Ublituximab	CD20	MS (phase II)
Obinutuzumab	CD20	(Case reports in MAG neuropathy)
Obexelimab	CD19 and FcRIIb	Not tried
Inebilizumab	CD19	MS (phase I), NMOSD (phase II/III, FDA-approved for NMO-SD)
		Under investigation in NMDA encephalitis (NCT04372615)
Bortezomib	Plasma cells	AQP4 NMOSD (series)
Daratumumab	CD38	AE (cases)
Efgartigimod	FcRn	MG (FDA-approved for MG)
Satralizumab	IL-6R	NMOSD (phase III, FDA-approved for NMO-SD)
Tocilizumab	IL-6R	NMOSD (series), MOGAD (series), NMDAR encephalitis
Evobrutinib	BTK	MS (phase II)
Tolebrutinib	BTK	MS (phase II)
Fenebrutinib	BTK	Under investigation in MS (NCT04586023, NCT04544449)

*MS* multiple sclerosis, *AQP4* aquaporin 4, *NMOSD* neuromyelitis optica spectrum disorders, *MOGAD* myelin-oligodendrocyte glycoprotein-associated disease, *AE* autoimmune encephalitis, *NMDAR* N-methyl-d-aspartate receptor, *MG* myasthenia gravis, *CIDP* chronic inflammatory demyelinating polyneuropathy, *DM* dermatomyositis, *PM* polymyositis, *SPS* stiff-person syndrome, *MAG* myelin-associated glycoprotein, *CD* cluster of differentiation, *IL* interleukin, *FcR* constant fragment receptor, *BTK* Bruton tyrosine kinase

### Multiple Sclerosis

#### Evidence of B Cell Involvement

In MS, no single, disease-characterizing pathogenic autoantibody has been identified to date. Although historically viewed as a T cell–mediated disease due to focusing on T cells in the CNS lesions and influenced by HLA DRB1*15:01-associated genetic risk or transfer of experimental autoimmune encephalomyelitis by encephalitogenic T cells, B cells have now evolved as important contributors to the pathogenesis of the disease as supported by several fundamental observations. First, a diagnostic hallmark of MS has been the presence of oligoclonal bands in the CSF and intrathecal IgG (but also to a lesser degree IgM and IgA) synthesis [54–56]. Second, B cells can be present in MS lesions and further, in several documented cases of primary and primarily secondary progressive MS (SPMS), activated B cells form meningeal germinal centers, where they follow the same differentiation pathways as in secondary lymphoid tissue [57], as depicted in Fig. 3. Although intrathecal immunoglobulin production and meningeal B cell follicles, as well as lesional deposition of immunoglobulin and complement, are not unique to MS, they do unambiguously support B cell involvement as discussed below [22, 58, 59]. Finally, B cells are necessary for marmoset EAE [60].

Several investigations have examined the nature of B cells and immunoglobulins in patients with MS. In CSF, B cells amount 5% of lymphocytes (whereas CD3 T cells ~ 70%), and a significant fraction of B cells are memory B cells and CD138 + CD19 + short-lived plasmablasts, whereas frank plasma cells are rarer [61]. Analysis of the CSF BCR repertoire and peptidome has shown significant overlap suggesting that oligoclonal bands are produced by CSF B cells [55]. Further, recombinant antibodies produced from CSF and lesional B cells were shown to target various intracellular antigens [62]. Analysis of the BCR repertoire of B cells from MS lesions, CSF, cervical lymph nodes, and peripheral blood has clearly shown the presence of related clones in all compartments [55, 63, 64]. Importantly, based on mutational analysis, B cells of the cervical lymph nodes were ancestral to lesional B cells.

In the peripheral blood, B cell subsets including memory B cells were not consistently numerically different compared to healthy controls [65–68]. Functional analyses however have shown that B cells of MS patients can produce less of the regulatory cytokine IL-10 but more of the pro-inflammatory GM-CSF, lymphotoxin α, TNF-α, and IL-6 [47, 51, 67, 69–71]. In relation to the BAFF/APRIL system, several studies point towards BAFF levels being normal in the serum of MS patients compared to controls and decreased in the CSF, although others found significantly increased levels [72–76]. In B cell–containing MS lesions, however, the expression of BAFF is upregulated in astrocytes proximal to BAFF-R-expressing immune cells. In addition, expression of BCMA (but not BAFF-R and TACI) is upregulated in MS lesions compared to the normal brain (54). Interestingly, BAFF may agonistically activate the Nogo receptor, which is upregulated in MS lesional astrocytes and microglia/macrophages [77] but can also be found on neurons and B cells [33, 78, 79]. This interaction may result in inhibition of axonal growth and potentially provide at least one of the missing links between immune responses and degeneration in MS patients.

### B Cell–Targeted Therapies in Multiple Sclerosis

The first B cell agent applied in MS was rituximab, a chimeric mouse/human anti-CD20 mAb that effectively depletes peripheral blood (but not lymphnode) B cells and a small subset of CD20 + T cells; it spares early pre-/pro-B cells and late plasma cells [1–3, 5, 80]. Notably, CD20^dim^ T cells can be found in all lymphatic organs; they are often CD8^+^ and some of them can be myelin-specific [80–82]. Cerebrospinal fluid B cells seem to be less affected by peripheral rituximab administration [83–85], although the drug itself can be detected in a very low concentration (up to 1000 times smaller than in the periphery) beyond the BBB [86].

**Fig. 4:**
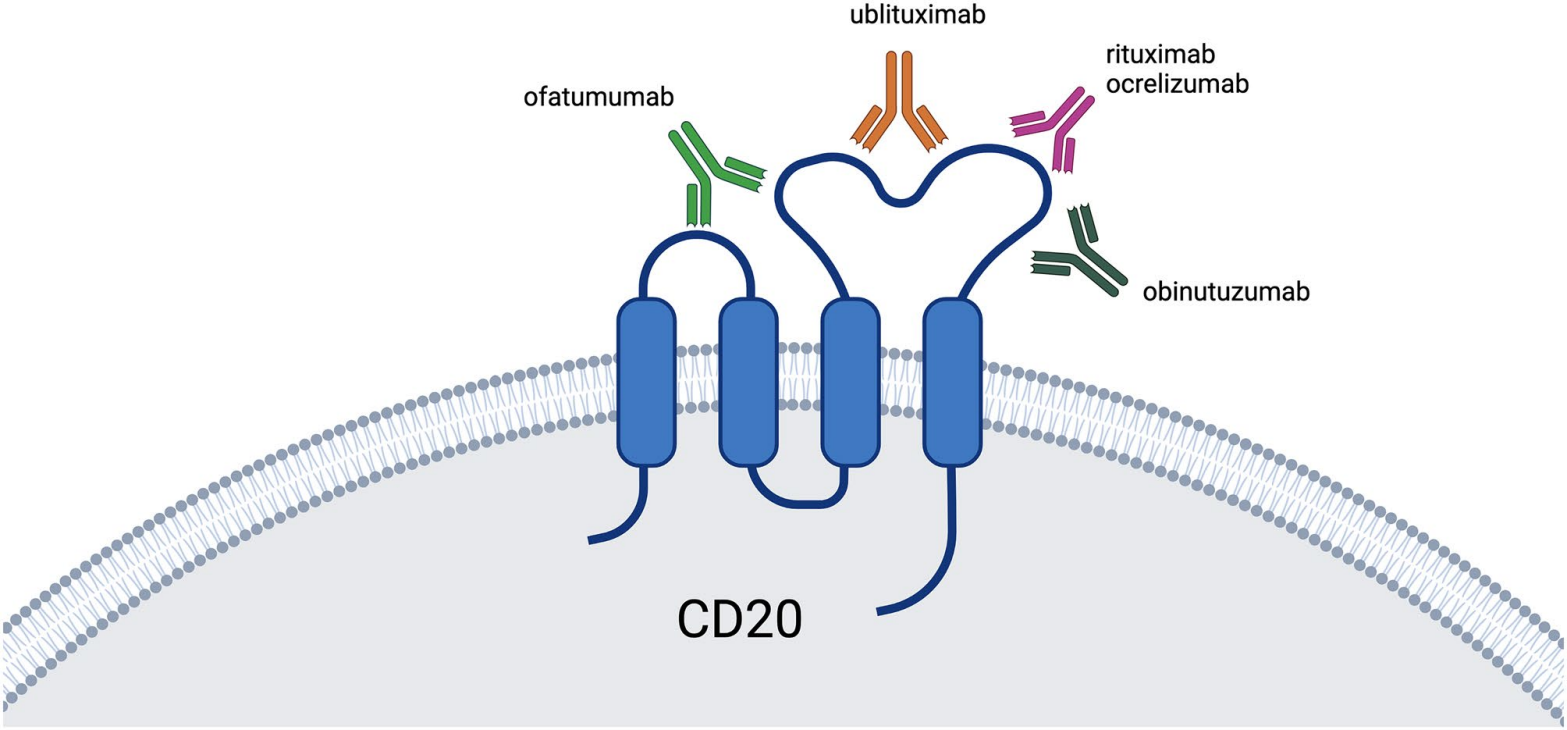
Binding sites of therapeutic CD20 monoclonal antibodies (mAbs). The human CD20 molecule consists of intracellular, transmembrane, and extracellular domains. The latter comprise two loops, a small and a bigger one. Different mAbs target different extracellular epitopes. Ofatumumab binds both loops; rituximab, ocrelizumab, ofatumumab, and ublituximab are type I antibodies, meaning that they can bind FcRIIb with their constant part and are therefore subject to internalization in contrast to obinutuzumab, the only type II mab

Phase 1 and 2 trials of rituximab in RRMS reported significantly reduced inflammatory brain lesions and a significant reduction in annualized relapse rates without significant serious adverse events [4, 87]. A phase 3 trial of rituximab in patients with primary progressive multiple sclerosis (PPMS) reported that, despite lack of significant differences in the primary endpoint, selective B cell depletion may affect disease progression in younger patients, particularly those with inflammatory lesions [88]. Two identical phase 3 trials of ocrelizumab, a fully humanized anti-CD20 antibody, in RRMS confirmed the great benefit of CD20 depletion with a reduction in relapse rate, disability progression, and an impressive 94% reduction of active MRI lesions compared to interferon beta-1α [89]. In a phase 3 PPMS trial, ocrelizumab was moderately effective in halting disability progression but total differences were driven, as in the case of rituximab, by the effect on MRI activity [90]. A 1-year, phase 2 trial of ublituximab, a novel chimeric anti-CD20 antibody targeting a distinct (in comparison to rituximab and ocrelizumab) CD20 epitope (Fig. 4), in 45 patients with RRMS showed promising results with 74% of patients achieving no evidence of disease activity [91]. Finally, two identical phase 3 trials of ofatumumab, a fully humanized anti-CD20 mAb administered subcutaneously, in patients with RRMS and SPMS with disease activity, showed a significantly decreased relapse rate, disability progression, MRI activity, and neurofilament light-chain levels, compared to teriflunomide [92]. Of note, ofatumumab targets different CD20 epitopes compared to rituximab, ocrelizumab, and ublituximab binding not only the large loop of CD20 but also the small loop closer to the B cell membrane (Fig. 4). As a result, B cell lysis is likely more effective.

Collectively, CD20 B cell depletion has become the mainstay of high efficacy therapy of MS, achieving an almost total control of MRI activity. CD20 depletion, although more immunosuppressive than natalizumab, an α4 integrin blocker that is also highly effective in RRMS [93], carries a far smaller risk of PML, with only 10 cases reported in the ~ 200,000 patients with MS that have received ocrelizumab post marketing. Of those, 9 were carry-over cases with previous use of natalizumab and fingolimod, while in one, a 78-year-old patient with MS and low absolute lymphocyte count prior to treatment initiation, this was likely due to immune senescence [94]. These observations have lent support to the practice applied in some centers of performing a lumbar puncture prior to the switch from natalizumab to rituximab in order to exclude subclinical PML. In contrast to natalizumab, discontinuation of CD20 B cell depletion therapy is not associated with rebound disease activity [95].

Dosage of CD20 depleting agents is the subject of an ongoing debate, particularly for long-term treatment. A very low dose (10 mg) of rituximab administered intrathecally can almost completely deplete CD20 B cells in the periphery [96], while a 100-mg iv infusion adequately depletes peripheral B cells for at least 6 weeks [97]. Standard regimens such as rituximab 1000 mg on days 1 and 15 and month 6 (adopted from rheumatoid arthritis) [98], or ocrelizumab 300 mg on days 1 and 15 and 600 mg every 6 months for 2 years (as applied in the phase 3 trial), usually deplete peripheral B cells between 6 and 12 months without at first affecting total IgG or antibody titers, e.g., against tetanus [99–101]. Administering 500 mg of rituximab whenever B cells or CD27 memory B cells rise above 1% is followed by some to determine the need for repeat dosing [100, 102]. As the effect of CD20 B cell depletion on disease activity may last longer than the biological effect on peripheral B cells, the pressure for close follow-up repeat dosing is not high [101]. Repeated dosing eventually lowers total IgG (increasing susceptibility for infection) [103, 104] and may affect a longer depletion of B cells. This dictates the need to monitor IgG serum levels before each follow-up infusion. Further, because dosing was not weight-adapted in the phase 3 ocrelizumab trial in PPMS, a post hoc analysis showed that patients who received doses higher in milligrams per kilogram of body weight showed less progression; this has sparked the initiation of high-dose ocrelizumab trials in both RRMS and PPMS (NCT04548999 and NCT04544436). Although B cell depletion therapies have been combined with other agents in lymphoma and in some MS patients with mitoxantrone and copaxone, CD20 depletion in MS and other neurological autoimmunities is used in isolation, a practice we also endorse for safety.

In addition to CD20 mAbs, B cell depletion can also be achieved with the targeting of CD19, a surface marker slightly more broadly expressed on the surface of B cells, both towards immature B cells and towards antibody-secreting cells [3]. One such humanized and afucosylated antibody is inebilizumab, which has been administered to patients with relapsing MS in the context of a dose- and route-finding, phase 1 trial. Importantly, the trial showed that CD20 B cell depletion was more long-lasting compared to anti-CD20 agents, with repopulation beginning at 36 weeks (later for higher doses and iv vs. sc route); further, inebilizumab administration trended towards reduced Gd-enhancing and new T2 lesions with an acceptable safety profile [105].

Bruton tyrosine kinase inhibitors (BTKIs) primarily target B cells but also microglia, the cells implicated in chronic active lesions and potentially in disease progression, a feature of MS not adequately controlled with CD20 depletion. In addition, BTKIs have the advantage of being oral agents and CSF-penetrant. Two dose-finding, phase 2 trials in MS have already been performed and multiple phase 3 trials are underway. A phase 2 trial of evobrutinib in patients with relapsing MS has shown significant reduction of contrast-enhancing MRI lesions at weeks 12 to 24 (although still detectable) and decrease of the annualized relapse rate compared to DMF [106]. Similarly, a phase 2 trial of tolebrutinib in patients with relapsing MS has shown a significant reduction of contrast-enhancing lesions at week 12; both evobrutinib and tolebrutinib were safe with no deaths or life-threatening adverse events [107].

Atacicept is a dimerized soluble TACI receptor fused to an Fc that competes with endogenous TACI, BAFF-R, and BCMA for BAFF and APRIL binding [41]. Atacicept improved SLE and RA, at least at the highest administered dose, but led to increased disease activity in MS at all doses despite decreasing immunoglobulin levels and mature naïve B cell counts, suggesting lack of pathogenic immunoglobulins in MS [108]. The reasons for the detrimental effect of atacicept include decrease of non-specific Fc blockade by immunoglobulins (in contrast, IVIg that upregulates FcRIIb is minimally, if any, effective in MS), elimination of B regulatory cells in the naïve B cell fraction (although these are also eliminated by CD20 depletion), disruption of BAFF-mediated Breg induction, and a stimulated increase of pathogenic memory B cells [41, 109, 110].

### Neuromyelitis Optica

#### Evidence of B Cell Involvement

In AQP4-NMO and -NMO spectrum disorders (NMOSDs), the implication of B cells in the pathophysiology of the disease is clearer than that in MS because of the presence of pathogenic, disease-characterizing, antibodies against AQP4. Analysis of serum and CSF AQP4 titers points to lack of intrathecal synthesis of AQP4 Abs [111]; cases of isolated CSF AQP4 Ab-positivity are very rare [112]. In addition, an experimental model with peripheral administration of a monoclonal AQP4 antibody has shown CNS entry of the antibody despite an intact BBB [113]. However, AQP4-specific plasmablasts have been located in the CSF [114] and shown to be related to peripheral blood plasmablasts and memory B cells [115, 116]. This is relevant to anti-B cell mAbs because they do not enter the CNS to a significant degree, in contrast to small molecules such as BTKIs that easily do.

Several lines of evidence implicate IL-6, a Th2 cytokine that promotes germinal center formation and antibody-secreting cell survival in AQP4-NMOSD. First, IL-6 m-RNA was significantly increased in all areas examined in an autopsied AQP4-NMOSD patient, particularly the optic nerve [117]. Second, patient-derived peripheral plasmablasts cultured in the presence of IL-6 have been shown to produce AQP4 autoantibodies [118]. Third, IL-6 is significantly elevated in a fraction of NMOSD patients [119–121]. Among the other B cell–related mediators, primarily BAFF, but also APRIL, have been found increased in the CSF of most NMO patients [72, 122, 123]. In the periphery, a single study reported a low proportion of IL-10-producing regulatory B cells in AQP4-NMO patients compared to healthy donors [123].

#### Anti-B Cell Efficacy

There is currently a large amount of evidence supporting the effectiveness of rituximab in treating AQP4-NMO but no clinical trials have been conducted. Open label studies report significant reduction in relapse rate although in some patients relapses may occur, even in the absence of B cells [124–130]. It is not uncommon for a relapse to occur shortly after rituximab initiation, prompting prednisone treatment for the first month. Despite improving relapse rates and reducing disease activity, AQP4-IgG titers are not consistently reduced even after long-term B cell depletion, pointing to the presence of long-lived plasma cells [131, 132]. In a cohort of 100 NMO patients treated with rituximab, a 96% reduction of annualized relapse rate compared to pre-rituximab treatment was noted [133].

Because up to 24% patients do not respond to rituximab [124], alternative therapeutic approaches targeting B cells have been explored. Since AQP4 antibodies need complement to exert pathogenicity [134], complement therapy with eculizumab has been shown to be very effective leading to an FDA-approved indication, but with prohibitive cost due to frequent dosing [135]. Additionally, a trial of 600 mg (on days 1 and 15) of inebilizumab, the CD19 mAb, in combination with 20 mg of prednisone for 14 days followed by a 7 day taper vs. placebo demonstrated significant efficacy in NMO patients (93% with AQP4 antibodies); in 197 days of follow-up, 21 of 174 (12%) treated patients relapsed vs. 22 of 56 (39%) in the placebo group [136]. As with CD20 depletion, a few patients did suffer relapses (optic neuritis and myelitis) under treatment despite successful B cell depletion.

A third therapeutic strategy, indirectly affecting B cell functions, includes agents targeting IL-6 or its receptor. In a phase 3 trial of satralizumab, a humanized mAb that binds both the soluble and the membrane-bound IL-6 receptor, 30% of patients with NMOSD (65–72% AQP4 antibody-positive) experienced relapses while on satralizumab monotherapy vs. 50% of patients receiving placebo monotherapy (*p* = 0.018) [137]. Moreover, in a case series of 8 NMO patients (6 AQP4-positive) with highly active disease activity despite treatment with rituximab, administration of tocilizumab, a humanized anti-IL-6 receptor antibody, resulted in relapse control in all patients [138]. Similarly, in a series of 4 AQP4-NMO patients, good clinical control was achieved in all patients [139]. Refractory NMO cases have also been treated with the proteasome inhibitor bortezomib, an agent originally used against antibody production in multiple myeloma. In a case series of 5 AQP4-NMO patients resistant to previous immunotherapy (in 2 cases rituximab), bortezomib resulted in relapse control during the 1 year of follow-up in 4 of 5 treated patients, along with reduction of AQP4 titer, CD19 B cells, and CD138 plasma cells [140]. Neuropathy, a common adverse effect with bortezomib, was not reported.

### Myelin Oligodendrocyte Glycoprotein Antibody–Related Disease

Myelin oligodendrocyte glycoprotein (MOG) antibody–related disease (MOGAD) has emerged from the body of NMOSD as a separate disease entity, because patients harboring MOG and AQP4 autoantibodies differ significantly [141]. Although pathogenicity of MOG antibodies is debated [134, 142], they do target a surface molecule in contrast to nonpathogenic autoantibodies targeting intracellular antigens. The pathogenicity of MOG autoantibodies is likely different in patients with high MOG autoantibody titers compared to patients with low titers [112, 142], which translates into potential pathogenicity of MOG-specific B cells. In children with monophasic acute demyelinating syndrome, which is often associated with MOG autoantibodies, CSF analysis has revealed IL-6 elevations that correlate with MOG antibody titer; moreover, CSF elevations of IFN-γ, IL-17, and IL-10 were also noted in a minority of patients [143]. A multiplexed analysis of 32 cytokines in the CSF of pediatric MOGAD patients showed that not only IL-6, but many cytokines, especially IFN-γ, TNF-α, G-CSF, GM-CSF, IL-17, BAFF, APRIL, IL-1, and IL-10, were elevated compared to patients with MOG-negative demyelinating disease and controls [144].

Controlled therapeutic trials in MOGAD, as in all rare disorders, are hard to perform. Case series data indicate that CD20 B cell depletion can be effective in MOGAD; however, refractory cases occur more frequently than in AQP4 NMO and relapses can be seen in up to 50% of patients (*n* = 121) despite efficient B cell depletion [124, 145, 146]. Anti-IL-6 therapy with tocilizumab in small case series of 3 and 10 patients has shown that treated patients remained relapse-free for an average of 23 to 28 months [139, 147]. Common side effects included neutropenia, low platelet count, and liver enzyme elevations apart from infections (https://www.ema.europa.eu/en/documents/product-information/roactemra-epar-product-information_en.pdf). Special note should be given to the observations that in uncontrolled series IVIg is particularly effective in MOGAD, especially in children, and it is currently the preferred therapy in patients who exhibit more than one relapse [148].

### Autoimmune Encephalitis

Over the past 10 years, several pathogenic or potentially pathogenic autoantibodies against surface synaptic proteins of the CNS have been linked to specific clinical presentations that fall within the general category of autoimmune encephalitis [149]. Accordingly, autoreactive, antigen-specific B cells are directly involved in the disease pathogenesis with evidence of intrathecal synthesis of autoantibodies and presence of antigen-specific B cells in the CSF in the more common subtypes of NMDAR and LGI-1 encephalitis [150–152]. Although recurrences can rarely occur, in the majority of NMDAR and LGI-1 cases, the disease is monophasic.

As in MOGAD, rarity of the disease makes controlled therapeutic trials difficult. In a case series of 14 patients with inadequate response to first-line immunotherapy (steroids, IVIg, and PLEX), clinical outcomes with second-line immunotherapy (rituximab plus cyclophosphamide) were favorable with 70% reaching a modified Rankin scale (mRS) of < 2 at 6 months [153]. Similarly, in a case series of 8 pediatric refractory NMDAR patients, rituximab monotherapy led to an mRS of < 2 in 60% [154]; this has been also our experience with 2 pediatric patients. It should be noted however that cyclophosphamide, although seemingly effective, carries a high risk of affecting reproductive ability that should be taken into account when treating young patients. In a large case series of 78 consecutive NMDAR-positive patients, a combined steroid, IVIg, rituximab, and tocilizumab regimen was overall advantageous compared to a steroid, IVIg, and rituximab regimen (and teratoma removal whenever applicable in both arms) [155]. Of note, a trial of CD19 depletion with inebilizumab in NMDAR encephalitis is currently ongoing (NCT04372615). In other antibody-positive autoimmune encephalitis with autoantibodies against surface and extracellular proteins or paraneoplastic antibodies targeting intracellular components, rituximab can be effective [131, 156]. Interestingly, the anti CD38 antibody daratumumab, targeting antibody-secreting cells (but also non-B cells such as monocytes and NK cells), has been effective in isolated, severe cases of autoimmune encephalitis with autoantibodies to caspr2 and NMDAR [157, 158].

### Chronic Autoimmune Polyneuropathies

In chronic autoimmune polyneuropathies including chronic inflammatory demyelinating polyneuropathy (CIDP), Multifocal Motor Neuropathy (MMN), and IgM anti-myelin-associated glycoprotein antibody demyelinating neuropathy (anti-MAG neuropathy), B cell involvement is supported by several pieces of evidence. Different anti-ganglioside and glycolipid antibodies, most often associated with GBS subtypes, may be pathogenic as they can induce conduction block and acute neuropathy [159–162]. Overall, IgG antibodies that react with GM1, GD1a, GalNAc-GD1a, and GM1b are found in 80% of patients with axonal GBS (AMAN and AMSAN), while anti-GQ1b antibodies are detected in more than 90% of patients with the Miller-Fisher variant. Anti-GM1 antibodies are also detected in 50% of MMN patents.

In contrast to GBS, however, no single antibody has yet been identified as the primary causative factor in CIDP, the most common chronic autoimmune neuropathy, in spite of the compelling indirect evidence provided by the beneficial effect of plasmapheresis [159, 163]. The first indication that antibodies are involved in CIDP was the presence of complement-fixing IgG and IgM deposits on the patient’s peripheral nerves and myelinated fibers [164] while the presence of an IgG band in their CSF provided further credence [165]. Exception to the absence of pathogenic autoantibodies in CIDP is the patients with autoimmune nodopathies comprising 10% of CIDP patients that harbor specific antibodies of IgG4 subclass targeting paranodal antigens at the nodes of Ranvier [166]. These antibodies, directed against paranodal antigens, such as neurofascin-155, CASPR1, and contactin-1, which are necessary for maintaining nodal structure, induce conduction block by affecting protein–protein interaction and cell adhesion [167, 168]. B cells from CIDP patients exhibit reduced expression of FcγRIIB, an inhibitory receptor that prevents B cells from entering the germinal centers to become IgG-positive plasma cells [169]. This observation further supports the role of B cells in the disease. Finally, serum levels of BAFF were found decreased in CIDP, although this may be confounded by the presence of anti-BAFF antibodies in the IVIg preparations that these patients had received for therapy.

Regarding MAG-associated neuropathy, strong evidence suggests that the antibodies are causative because (a) IgM and complement are deposited in myelinated fibers in patients’ sural nerve biopsies [170]; (b) the patients’ IgM co-localizes with MAG on the areas of the split myelin implicating a myelin disadhesion process induced by the circulating anti-MAG IgM [171]; (c) in skin biopsies from the patients, there is deposition of IgM, complement, and MAG on the intradermal myelinated fibers with a concurrent loss of nerve fibers suggesting IgM-induced fiber loss [171]; and (d) of corroborative data from animal models, either from intraneural injections into peripheral nerve or from immunization with sulfoglucuronyl paragloboside, a glycolipid cross-reactive with MAG [172].

In terms of treatment, CD20 depletion has been applied in CIDP [173] but no controlled studies have been conducted. Anti-B cell depletion therapy with rituximab is however especially successful in CIDP patients with paranodal antibodies which are of IgG4 subclass, as discussed below [174, 175]. Patients with anti-MAG neuropathy treated with rituximab have seen clinical benefit, reduction of anti-IgM and anti-MAG antibodies, and Treg upregulation [16, 176]. Two controlled trials however have not shown statistically significant results due to variability of the clinical phenotype [16, 177]. Nevertheless, experience indicates that 40% of these patients respond to rituximab, which is the treatment of choice as these patients do not respond to IVIg, PLEX, and steroids [178]. Further mechanistic investigations have shown that patients with anti-MAG neuropathy harbor substantial clonal expansions of IgM memory B cells that recognize MAG, while patients who do not experience clinical improvement after rituximab have higher numbers of clonal anti-MAG memory B cells before and after therapy and lower somatic hypermutation frequencies of IgM memory B cells [179]. Obinutuzumab, an anti-CD20 antibody that targets a different epitope than the other anti-CD20 mAbs, as depicted in Fig. 4, is also resistant (in contrast to rituximab) to internalization by B cells through FcRIIbeta, thereby achieving more effective depletion in theory [5]. Obinutuzumab was tried in 2 anti-MAG-positive patients unresponsive to rituximab and was ineffective in our hands [180]; however, others found some benefit [181]. In MMN, rituximab can be effective in anecdotal series of patients insufficiently responding to IVIg.

### Stiff-Person Syndrome and Progressive Encephalomyelitis with Rigidity and Myoclonus

In stiff-person syndrome (SPS) and SPS-spectrum disorders (SPS-SD) as well as patients with progressive encephalomyelitis with rigidity and myoclonus (PERM), B cell involvement is supported by the presence of seemingly nonpathogenic antibodies against glutamic acid decarboxylase [182, 183], an intracellular antigen, or by the likely pathogenic antibodies against the glycine receptor [184]. In addition, both memory B cells and bone marrow plasma cells specific for GAD have been shown to be present in patients with SPS and have the ability to produce GAD antibodies upon non-specific stimulation (memory cells) but also without stimulation (plasma cells) [185, 186]. Case reports have provided evidence that rituximab can help patients with stiff-person syndrome [187, 188]. In the largest controlled study we have conducted, however, rituximab was not statistically effective because of a strong placebo effect but 35% of patients clinically improved and some of them with impressive benefits [189]. Rituximab still remains a treatment option in SPS patients unresponsive to IVIg; further, in a recent case series, rituximab improved some SPS patients [190]. Rituximab has been also effective in a glycine receptor antibody-positive PERM patient of ours [191]. This patient, hospitalized in the ICU for 12 months, requiring mechanical ventilation and being IVIg-unresponsive, impressively improved after rituximab infusion to the point of being able to walk and be discharged; interestingly, improvement was associated with significant reduction of the glycine receptor antibodies in his serum and disappearance from the CSF.

### Myasthenia Gravis

In myasthenia gravis (MG), B cells are primarily involved in antibody production against the AChR (primarily IgG1 subclass antibodies) and muscle-specific kinase (MuSK, primarily IgG4 subclass antibodies) [192, 193] but potentially also in antigen presentation given their BCR specificity and T cell involvement [194–197]. One distinguishing feature between the AChR and MuSK subtype is that the autoantibody-producing cells tend to be short-lived in MuSK and long-lived in AChR MG [131]. In addition, BAFF levels have been found increased in active disease [198]. Because of the central role of B cells in the pathophysiology of the disease, depleting or suppressing B cell function can restore immune balance and result in clinical improvement.

Rituximab has been tried in patients with MG whose disease was difficult to manage with the conventional therapies. In MuSk MG, with autoantibodies being of the IgG4 subclass, a pronounced and long-lasting remission is characteristic, coinciding with reduction or even disappearance of antibody titers [99, 199, 200] due to depletion of MuSK autoantibody-producing short-lived plasma cells. In AChR MG, several case series support a benefit of rituximab [200–203]. A relatively small phase 2 trial failed to meet the primary endpoint, mostly due to study design (NCT02110706). In a recent, important, large prospective study of 72 generalized MG patients [204] with new-onset disease, early treatment led to early remission without the need for any maintenance therapies; the patients treated at a later time also improved having fewer relapses and not requiring rescue therapy for at least 24 months. The fact that rituximab can induce early remission if used early without the need for long-term maintenance treatment has significant implications in clinical practice especially in the context of being safer than the conventional immunosuppressants. A phase 2 trial of the BAFF antagonist belimumab, an agent already approved for the treatment of SLE, was negative in MG [205]. In contrast, a phase 3 trial of the complement antagonist eculizumab met all the secondary endpoints and the antibody was approved for the treatment of refractory MG [206]. Similar has been the effect of efgartigimod, an FcRn inhibitor, which by enhancing the catabolism of all IgG, including the circulating AChR antibodies, led to significant clinical improvement in a large phase III clinical trial [206]; this study has now led to FDA approval. These last two drugs that target AChR antibodies and their function may change the treatment algorithm in MG patients in the near future, if economics is not a practical issue [200].

### Inflammatory Myopathies

In all the main categories of inflammatory myopathies (dermatomyositis (DM), necrotizing autoimmune myositis (NAM) and inclusion body myositis (IBM), B cells and plasma cells are present in muscle tissues [3, 207, 208]. Immunoglobulins are also deposited on endomysial capillaries in DM and complement is activated in DM and NAM [209]. It is however important to note that the inflammatory infiltrates consists of other immune cells as well, such as CD8 T cells in IBM and macrophages in NAM. A second argument for the involvement of B cells in the mechanism of inflammatory myopathies is the presence of autoantibodies, although not pathogenic, targeting intracellular molecules. It is therefore compelling that B cell–depleting therapies using anti-CD20 mAbs have been quite promising [210, 211].

Specifically, evidence provided by case reports and open label studies for the effectiveness of rituximab in the treatment of inflammatory myopathies is encouraging, reporting up to 75% response rates [212]. In a study of juvenile DM (48 patients) and adult PM (75 patients), a few indicators including the presence of antisynthetase and anti-Mi-2 antibodies and lower disease damage might predict, as claimed by the authors, clinical improvement after rituximab treatment [210]; the inclusion however of 75 patients with PM, which is a very rare disease -if it exists-is of concern. Finally, in a study of 200 DM/PM patients that were randomized to rituximab early and rituximab late, there was no difference between the groups; however, at week 44, when all the patients had received rituximab, 83% met the definition of improvement [213].

### Autoimmune Neurological Disorders with IgG4 Antibodies

The main IgG4 antibody–mediated neurological disorders (IgG4-ND) include MuSK myasthenia; CIDP with nodal/paranodal antibodies to Neurofascin-155, contactin-1/caspr-1 or pan-neurofascins; anti-LGI1 and CASPR2-associated limbic encephalitis, Morvan syndrome, or neuromyotonia; and possibly anti-IgLON5-spectrum CNS disease [214]. These disorders are distinct due to unique functions of IgG4 antibodies which exert pathogenic effects on their targeted antigens by blocking enzymatic activity or disrupting protein–protein interactions affecting signal transduction pathways. In contrast to IgG1 subclass, the IgG4 antibodies do not largely activate complement or bind to inhibitory FcγRIIb receptor and cannot engage in cross-linking of the targeted antigen with immune complex formation and endocytosis. Because the IgG4 antibodies do not trigger inflammatory processes, the conventional anti-inflammatory therapies, especially with IVIg and steroids, are ineffective or not sufficiently effective in inducing short-term or long-term remissions [214, 215]. Data from large series of patients with MuSK-myasthenia and CIDP with nodal/paranodal antibodies indicate that these patients robustly respond to B cell depletion therapy with rituximab which, by targeting memory B cells and some IgG4-producing CD20-positive short-lived plasmablasts, exerts long-lasting clinical remissions. Similar benefits are noted in a small anectodal series of patients with anti-LGI1 and CASPR2-associated limbic encephalitis. In IgG4-ND, other anti-B cell agents that target CD19/20, especially those that concurrently activate the inhibitory FcγRIIb receptors (which IgG4 antibodies cannot bind to) thereby affecting functional blockade of CD19 without cell lysis such as obexelimab, can be even more beneficial as proposed in IgG4-related disease (NCT02725476) [214, 215].

## Biomarkers of Clinical Response to B Cell Depletion Therapies

Although CD20 depletion is actively used for treating many autoimmune neurological diseases, a useful, easy-to-use biomarker of response to treatment is lacking. The biological effect is typically monitored by flow cytometric assessment of the CD19 B cell count, with the rationale being that the mAb used for the count should not have potentially overlapping epitopes with the therapeutic antibody. As with other drugs such as interferons, natalizumab, and anti-TNF agents, anti-drug antibodies can develop and correlate with incomplete B cell depletion [216]. In many cases, treatment efficiency correlates with peripheral B cell depletion and effective depletion of the CD20 + CD27 + (IgD-) memory B cells in particular [217], which is mostly currently used to monitor the need for the next infusion [217]. In a small number of patients with anti-MAG neuropathy, clinical improvement with rituximab appears to coincide with the reduction in total and MAG IgM, possibly attributed to depletion of memory cell precursors of short-lived antibody secreting cells [16]. The same is true for MuSK MG [99, 218]. In the MAG study, the patients showing no clinical improvement after rituximab were distinguished from responders by a higher load of clonal IgM memory B cell expansions before and after therapy as well as by persistence of clonal expansions despite efficient peripheral B cell depletion, and by lack of substantial decrease in somatic hypermutation frequencies of IgM memory B cells [179]. These results were however based on a small number of patients. In a study of pemphigus patients (which harbor IgG4 autoantibodies against desmoglein), significant and prolonged clinical response to rituximab was observed in the majority of patients and was connected to a persistently high proportion of transitional, IL-10-secreting naïve B cells in the reconstituted B cell fraction [17]. Similar relative elevations of transitional B cells have been observed in NMO patients treated with rituximab [219]. On the other hand, rituximab failed to restore B cell tolerance defects despite clinical response in three out of three patients with type 1 diabetes [220].

In AQP4 NMO, where a relapse is more likely to result in a permanent deficit than in MS and disease activity is more likely to return with B cell repopulation—in particular memory B cell repopulation [101, 124]—close monitoring of CD19 + and CD19 + CD27 + B cell depletion and reconstitution is used as a tool for relapse prevention and timely treatment, further improving the treatment benefit-risk ratio [125]. Several studies showing that the repopulation of peripheral blood by B cells, especially memory B cells, coincides with clinical relapses support the practice of close CD19 + and CD19 + CD27 + memory B cell monitoring by flow cytometry in these patients [124, 125, 217, 219, 221, 222]. In MOGAD, however, few relapses are connected to memory B cell reappearance, so their monitoring does not seem to be consistently helpful [124]. Furthermore, relapses in MOGAD can occur in several cases under complete B cell depletion [146].

Overall, it is conceivable that monitoring transitional/regulatory B cells pre- and post-treatment along with memory B cells could provide a comprehensive way for evaluating efficacious therapy. In addition, genetic markers such as FcγRIIIA polymorphisms in anti-MAG neuropathy and FCGR3A polymorphisms in NMO or generally studying the B cell receptor repertoire may prove to be of value [133, 223, 224]. Finding more sensitive and specific biomarkers will be important in determining the frequency of infusions needed to prevent relapses or inducing long-lasting remissions and lead to personalization of B cell depletion therapies. In IgG4 neurological diseases, the depletion or reappearance of the noted pathogenic IgG4 antibodies—not the total IgG4 level—seems to be an easy, highly promising, biomarker as proposed [214].

## The New Era of B Cell-Biological Therapeutics

Novel therapies that specifically target elements of the immune system, B cells, T cells, and various receptors have revolutionized the field of immunotherapies constituting a paradigm shift from previous generation therapies that suppressed or modulated the immune system indiscriminately [200]. These specific therapies, often developed for hematological malignancies, have found their niche in the effective management of difficult-to-treat neurological diseases. As new data emerge, almost on a daily basis, regarding the pathophysiology of autoimmune neurological diseases, clinicians are becoming increasingly able to select the most appropriate therapy for treating a specific disease. Innovative research in the field of neurotherapeutics not only aims to develop more potent drugs, i.e., drugs that eliminate more efficiently a particular cell population or that are better tolerated and safer, but also to identify specific biomarkers informative for selecting and monitoring the most appropriate therapy. In the field of B cell therapeutics, the progress made has been impressive with more than 4 drugs already approved and others in the offing. Future research should focus on drugs that may also target antibody secreting cells, drugs that may not affect B regulatory cells, and drugs that selectively deplete pathogenic, antigen-specific B cells. Anti-B cell agents like obexelimab, a bispecific antibody that targets both CD19 and FcγRIIB, are especially attractive because they functionally inhibit B cells [214]. Importantly, how these indices correlate to clinical improvement on a single patient basis will be very useful for the tailor-made therapies of precision medicine.

## Supplementary Information

Below is the link to the electronic supplementary material.

Supplementary file1 (PDF 631 kb)

Supplementary file2 (PDF 1071 kb)
