# Supplementary material for: Evolution of Anti-B Cell Therapeutics in Autoimmune Neurological Diseases
Source: Neurotherapeutics. 2022 Feb 18;19(3):691–710. doi: 10.1007/s13311-022-01196-w (PMC9294112; doi:10.1007/s13311-022-01196-w)
Supplement: Supplementary file 1 — Supplementary file1 (PDF 631 kb) [file 13311_2022_1196_MOESM1_ESM.pdf]

INTERNATIONAL COMMITTEE of MEDICAL JOURNAL EDITORS

ICMJE Form for Disclosure of Potential Conflicts of Interest

Section 1. Identifying Information

1. Given Name (First Name)
Marinos

2. Surname (Last Name)
Dalakas

3. Effective Date (07-August-2008)
29-November-2021

4. Are you the corresponding author? ☑ Yes ☐ No

5. Manuscript Title
Evolution of anti-B-cell therapeutics in autoimmune Neurological diseases

6. Manuscript Identifying Number (if you know it)
NERX-D-21-00572

Section 2. The Work Under Consideration for Publication

Did you or your institution at any time receive payment or services from a third party for any aspect of the submitted work (including but not limited to grants, data monitoring board, study design, manuscript preparation, statistical analysis, etc...)?

Complete each row by checking "No" or providing the requested information. If you have more than one relationship click the "Add" button to add a row. Excess rows can be removed by clicking the "X" button.

The Work Under Consideration for Publication

Type	No	Money Paid to You	Money to Your Institution*	Name of Entity	Comments**	

* This means money that your institution received for your efforts on this study.
** Use this section to provide any needed explanation.

Section 3. Relevant financial activities outside the submitted work.

Place a check in the appropriate boxes in the table to indicate whether you have financial relationships (regardless of amount of compensation) with entities as described in the instructions. Use one line for each entity; add as many lines as you need by clicking the "Add +" box. You should report relationships that were present during the 36 months prior to submission.

Complete each row by checking "No" or providing the requested information. If you have more than one relationship click the "Add" button to add a row. Excess rows can be removed by clicking the "X" button.

Relevant financial activities outside the submitted work

ICMJE Form for Disclosure of Potential Conflicts of Interest

Relevant financial activities outside the submitted work

Type of Relationship (in alphabetical order)	No	Money Paid to You	Money to Your Institution*	Entity	Comments	
Type of Relationship (in alphabetical order)	No	Money Paid to You	Money to Your Institution*	Entity	Comments	
1. Board membership	☐	☑	☐	Serves as DSMB chair for Octapharma in a study in CIDP		×
1. Board membership	☐	☑	☐	Editorial Board as Associate Editor for Neurology (N2) and TAND		× ADD
2. Consultancy	☐	☑	☐	Argenx, Alexion, Dysimmune Diseases Foundation, Grifols, Elsevier		× ADD
10. Payment for development of educational presentations	☐	☑	☐	Neuro-edu		× ADD

* This means money that your institution received for your efforts.
** For example, if you report a consultancy above there is no need to report travel related to that consultancy on this line.

Section 4. Other relationships

Are there other relationships or activities that readers could perceive to have influenced, or that give the appearance of potentially influencing, what you wrote in the submitted work?

☑ No other relationships/conditions/circumstances that present a potential conflict of interest

☐ Yes, the following relationships/conditions/circumstances are present (explain below):

At the time of manuscript acceptance, journals will ask authors to confirm and, if necessary, update their disclosure statements. On occasion, journals may ask authors to disclose further information about reported relationships.

Show All Table Rows   SAVE
